# Seeking synergies: understanding the evidence that links menstrual health and sexual and reproductive health and rights

**DOI:** 10.1080/26410397.2021.1882791

**Published:** 2021-02-18

**Authors:** Lucy C Wilson, Kate H Rademacher, Julia Rosenbaum, Rebecca L Callahan, Geeta Nanda, Sarah Fry, Amelia C L Mackenzie

**Affiliations:** a Independent Consultant, Rising Outcomes, Hillsborough, NC, USA. *Correspondence:* lucy.wilson@gmail.com; b Senior Technical Advisor, Product Development & Introduction, FHI 360, Durham, NC, USA; c Senior WASH Behavior Change and Integration Specialist, FHI 360, Washington, DC, USA; d Associate Director, Product Development & Introduction, FHI 360, Durham, NC, USA; e Scientist, Maternal and Child Health, FHI 360, Washington, DC, USA; f Senior Hygiene and School WASH Advisor, USAID WASHplus Project, FHI 360, Washington, DC, USA; g Scientist, Product Development and Introduction, FHI 360, Durham, NC, USA

**Keywords:** menstrual health, menstruation, reproductive health, contraception, gender norms

## Abstract

Global efforts to improve menstrual health and sexual and reproductive health and rights (SRHR) are fundamentally intertwined and share similar goals for improving health and well-being and increasing gender equality. Historically, however, the two fields have operated independently and missed opportunities to build upon their biological and sociocultural linkages. Biological touchpoints connecting the two fields include genital tract infections, menstrual disorders, contraception, and menopause. From a sociocultural perspective, intersections occur in relation to the experience of puberty and menarche, gender norms and equity, education, gender-based violence, and transactional sex. We describe evidence linking menstrual health and SRHR and offer recommendations for integration that could strengthen the impact of both fields.

## Résumé

Les efforts mondiaux pour améliorer la santé menstruelle et la santé et les droits sexuels et reproductifs sont fondamentalement indissociables et partagent des objectifs similaires pour améliorer la santé et le bien-être tout en accroissant l’égalité entre hommes et femmes. Traditionnellement, néanmoins, les deux domaines ont opéré de manière indépendante et ont manqué des occasions de s’appuyer sur leurs liens biologiques et socioculturels. Les points de contact biologiques reliant les deux domaines incluent les infections de l’appareil génital, les troubles menstruels, la contraception et la ménopause. Dans une perspective socioculturelle, les intersections se produisent en rapport avec l’expérience de la puberté et des première règles, les normes de genre et l’égalité entre hommes et femmes, l’éducation, la violence sexiste et le commerce du sexe. Nous décrivons les données liant la santé menstruelle avec la santé et les droits sexuels et reproductifs et proposons des recommandations pour une intégration susceptible de renforcer l’impact des deux domaines.

## Resumen

Los esfuerzos mundiales por mejorar la salud menstrual y la salud y los derechos sexuales y reproductivos (SDSR) están entrelazados fundamentalmente y tienen objetivos similares en común para mejorar la salud y el bienestar e incrementar la igualdad de género. Sin embargo, históricamente, los dos campos han operado de manera independiente y han perdido oportunidades de aprovechar sus vínculos biológicos y socioculturales. Los puntos de contacto biológicos que conectan los dos campos son: infecciones del tracto genital, trastornos menstruales, anticoncepción y menopausia. Desde la perspectiva sociocultural, ocurren intersecciones con relación a la experiencia de pubertad y menarquia, normas y equidad de género, educación, violencia de género y sexo transaccional. Describimos la evidencia que vincula la salud menstrual y SDSR, y ofrecemos recomendaciones para integración que podrían fortalecer el impacto de ambos campos.

## Introduction

Efforts to improve menstrual health (MH) and sexual and reproductive health and rights (SRHR) are fundamentally intertwined and aim to achieve similar overarching goals; both aim to facilitate greater access to information, products, and services to support practices and behaviours that lead to improved health and well-being and greater gender equality. Thus far, however, the common purpose and potential synergies between these two fields have gone largely unexplored.

Important international SRHR guidance and resources neglect to robustly include MH. For example, the recent Guttmacher-*Lancet* Commission on SRHR mentions MH only once in its 43-page report and does not include MH in its list of essential SRHR services.^1^ Similarly, the list of SRHR topics on the World Health Organization (WHO) website does not include MH.^2^ And with few exceptions, the programmes and research supported by the largest funders of global SRHR programmes do not address MH.^3^

In this paper, we argue that MH must be part of an expanded definition for SRHR and that work across the two fields should be more closely integrated. We describe the biological and sociocultural linkages between MH and SRHR, highlight key evidence and evidence gaps, identify entry points for programmes to offer integrated services, and offer recommendations for collaboration and synergy. While the work of improving SRHR and MH, and thus the need for better integration, is global, we have focused this paper on efforts in low- and middle-income countries (LMICs), primarily in Africa and Asia.

In addition, we appreciate efforts from within the MH field to embrace a gender-neutral approach that is inclusive of trans* and/or gender queer individuals who menstruate. We use gender-inclusive language, including “menstruators”, whenever possible. While we advocate for the inclusion of people of all gender identities in discussions of MH and SRHR, we also use “women and girls” in this article, especially when it is used in the primary literature. In addition, neither MH nor SRHR can be adequately addressed without attention to the gender norms and dynamics experienced by individuals in the cultures and communities in which they live, and thus we use gender-specific language when discussing those topics.

## Background on MH and SRHR

### Menstrual health

Every day, millions of people around the world struggle to manage their menstruation safely, effectively and with dignity. Combined with other menstrual-related challenges, such as pain, stigma and social restrictions, menstruation can have profound and detrimental impacts on health, education, economic opportunities, social engagement and overall well-being.^4–6^ The MH field, largely spearheaded by the water, sanitation, and hygiene (WASH) and education sectors, works to address MH challenges by expanding access to affordable, high-quality materials to collect or absorb menstrual blood, and safe and private toilet facilities with adequate water and soap, as well as information and education on menstruation and puberty.^7^ Despite broader need, MH efforts generally focus on very young adolescents aged 8–14 years, who are at or near menarche. While girls are the primary audience, the importance of educating and engaging children of all genders, parents, school teachers and administrators, as well as broader communities, is increasingly recognised.^8^ Attention to MH needs of adults, including in the workplace, has also grown.^6^

This work was previously framed as menstrual hygiene management (MHM), but the field has recently embraced the broader term *menstrual health,* or *menstrual health and hygiene.* Menstrual health includes not only menstrual hygiene and the management of menstruation, but also related issues such as pain, stigma and taboo, gender, understanding of the menstrual cycle and overall well-being. MH also more broadly frames the issue as a right to dignified menstruation.^9^

### Sexual and reproductive health and rights

According to the Guttmacher-*Lancet* commission, sexual and reproductive health is a “state of physical, emotional, mental, and social well-being in relation to all aspects of sexuality and reproduction, not merely the absence of disease, dysfunction, or infirmity”.^1^ The commission goes on to state: “Achievement of sexual and reproductive health relies on the realization of sexual and reproductive rights”, which include the rights to bodily integrity and personal autonomy; to define one’s own sexuality and gender identity; to decide whether, when, and with whom to be sexually active, to marry, and to have children; and to have the information, resources, support, and self-esteem necessary to fulfil those rights. SRHR services include those related to contraception and family planning, abortion, sexually transmitted infections, gender-based violence, maternal health, infertility, and reproductive cancers. Women of reproductive age, defined by the WHO as those who are ages 15–49 years, are generally the focus of SRHR services, but people of all genders are also increasingly engaged by SRHR programmes.^1^

It is worth noting the target age groups of most MH and SRHR programmes do not align. MH programmes typically work with very young adolescents, among whom discussion of other SRHR topics may be considered by some to be inappropriate or premature. Meanwhile, the SRHR field seems to assume knowledge of menstruation and its management has already been addressed in its population of interest. While some SRHR programmes include work with very young adolescents, these are usually stand-alone programmes and may be post-menarche for some menstruators.^10,11^ The siloed approach of the two fields results in a gap in meeting needs across a person’s life course.

While the challenges of managing menstruation may be hardest for adolescents when they first start menstruating, menstruation and MH concerns occur throughout the reproductive years. Data from the Performance Monitoring and Accountability (PMA) 2020 surveys conducted in 11 African countries show the extent to which individuals have access to materials to manage menstruation does not vary significantly by age. In most of these countries the majority of survey respondents, across their reproductive years (15–49 years), did not have everything they needed to manage their menstruation adequately.^12^

We are not the first to advocate for greater linkages between the MH and SRHR fields. Hennegan et al.^13^ have recently called for increased collaboration, although they primarily focus on linkages between MH and family planning and on programmatic integration recommendations. A technical brief from Population Services International also calls for increased integration of MH within SRHR, providing programmatic recommendations based on the evidence and existing efforts.^14^

In this paper, we provide a more detailed description of the biological and sociocultural linkages between MH and the broad field of SRHR. This framing offers support for integration and potential entry points for integrated programmes and services. While current evidence supports integration, we do acknowledge there are certain areas where the evidence is limited, weak, and/or mixed, and in some cases, the causal pathways underlying the linkages are uncertain.

## Biological linkages between MH and SRHR

In essence, menstruation is about reproduction. Menstruation is the *biological* indicator that pregnancy has not occurred. The egg that was released from the ovary was not successfully implanted in the uterus, and the uterine lining is being shed. This monthly process, the menstrual cycle, is central to human reproduction.

### Genital tract infections

Challenges related to “period poverty” or the inability to afford and/or otherwise access an adequate quality and/or quantity of menstrual products exist globally. Evidence from LMICs indicates that when menstruators cannot afford or access menstrual products, they often resort to improvised materials such as improperly cleaned or scavenged cloth or other materials such as leaves or tissue paper. They may also struggle to properly clean, dry and store reusable menstrual products if adequate clean water, soap and appropriate facilities are not accessible.^5,15^ A systematic review published in 2013 by Sumpter et al. stated, despite inconsistencies in the data, that there is strong reason to believe poor menstrual hygiene practices may cause reproductive tract infections, such as bacterial vaginosis and vulvovaginal candidiasis.^16^ A 2018 study in India came to a similar conclusion.^17^ Both bacterial vaginosis and vulvovaginal candidiasis are associated with increased incidence of pre-term birth^18^ and may increase susceptibility to HIV infection.^19,20^ Bacterial vaginosis may also lead to infertility^21^ and is associated with an increased susceptibility to chlamydia and gonorrhea.^22^ A positive association also exists between bacterial vaginosis and human papillomavirus.^23^

A 2016 study on the effect of improved menstrual hygiene on health outcomes among school girls in western Kenya found that the provision of menstrual cups, but not disposable menstrual pads, lowered rates of bacterial vaginosis. Meanwhile, the provision of both menstrual cups and disposable pads was associated with lower prevalence of sexually transmitted infections. The authors suggest that these results may be due to a reduction in girls engaging in transactional sex to obtain menstrual products.^24^

### Menstrual disorders

Menstrual disorders, such as dysmenorrhea and menorrhagia (defined below), affect general and reproductive health in many ways. These conditions may also limit an individual’s ability to engage in daily activities.^25,26^

Dysmenorrhea is a condition in which menstruation is accompanied by pain. Global literature reviews have found that the condition affects up to 91% of menstruators, with severe pain in 2-29% of them.^27,28^ For example, in a study among 880 secondary school students in Tanzania, 74% reported dysmenorrhea and 24% reported missing school as a result.^29^

Another menstrual disorder is menorrhagia, or heavy menstrual bleeding. Menorrhagia affects approximately 10-–30% of menstruators worldwide.^30,31^ Through menstruation, individuals with average cycles lose 0.5–0.68 mg of iron per day,^32^ and those with menorrhagia lose at least twice as much iron per day. Up to two-thirds of those with menorrhagia also have iron-deficiency anaemia.^31,33^ Adequate iron is essential to health, especially healthy pregnancies. Anaemia is a major contributor to maternal mortality^34^ and is also associated with adverse birth outcomes for neonates.^35^

A global systematic review found abnormal uterine bleeding, including menorrhagia, to also broadly impact health-related quality of life. Abnormal bleeding affects physical and emotional functioning, such as work productivity, and several studies in the review found that individuals with menorrhagia regularly missed work.^30^ Missing work and/or school is also common among those suffering from dysmenorrhea, with similar negative impacts on productivity. Dysmenorrhea can also have a negative effect on relationships, sleep quality, stress levels, and psychological health.^27^ As menorrhagia and dysmenorrhea often overlap, these quality-of-life effects may be related to blood loss, pain, or a combination of the two.

Endometriosis is a painful menstrual disorder in which endometrial tissue grows outside of the uterine cavity. The condition often goes undiagnosed but is believed to affect between 2% and 17% of menstruators worldwide. For some, it may be the underlying cause for dysmenorrhea.^36,37^ A strong correlation exists between endometriosis and infertility.^38^ Endometriosis can also lead to decreased productivity and engagement in school, work and the household,^37^ as well as dyspareunia (pain during sexual intercourse) and diminished quality of social and intimate relationships.^36^

Heavy or irregular bleeding and/or menstrual pain may signal endometriosis or another underlying condition, such as uterine fibroids, adenomyosis, gynaecological cancer, polycystic ovarian syndrome, or a blood clotting disorder. As such, access to care and related patient-provider communication about menstrual patterns that are troublesome or seem abnormal can be important in diagnosis and improving health.^25^ Clinical guidance in the United States recommends that health care providers ask adolescents about their menstrual patterns as a “vital sign” and means to screen for potential health concerns.^39^

### Role of contraception in alleviating menstrual disorders

While menstrual disorders can have detrimental impacts on reproductive and general health and well-being, contraception can play an important role in alleviating these disorders. Most hormonal contraceptive methods can serve as effective treatments for dysmenorrhea, menorrhagia, and endometriosis.^27,33,40^ Contraceptives may be the preferred treatment for some menstrual disorders, particularly as they preserve future fertility, unlike alternatives such as hysterectomy, and may be more cost-effective.^41^ In addition, a systematic review published in 2013 found that the levonorgestrel intrauterine device (LNG IUD), may offer a treatment option for those who are borderline anaemic.^42^

Prior to its approval as a contraceptive in the United States in 1960, the first oral contraceptive pill (Enovid) was initially approved and marketed as a treatment for menorrhagia and dysmenorrhea. Today, many people continue to use contraceptive methods as treatments for these conditions. A review of US data showed that 14% of oral contraceptive pill users did so *only* for non-contraceptive reasons and 58% of users did so at least in part for non-contraceptive purposes. The most commonly cited non-contraceptive purposes were to alleviate menstrual pain (31%) and to regulate menstrual cycle timing and duration (28%).^43^

### Contraceptive-induced menstrual changes

All hormonal contraceptives, as well as the non-hormonal copper-bearing IUD, affect menstruation, with variations in menstrual effects among individuals and within individuals over time. Contraceptive methods can cause changes in bleeding duration, volume, frequency and predictability; they may also affect uterine cramping, pain, mood, and other menstrual cycle symptoms. Combined hormonal methods, including combined oral contraceptives (COCs), generally result in more predictability and lighter bleeding.^44^ Progestin-only methods, such as implants, injectables, and the LNG IUD, also generally result in a lightening of menstruation. Most users experience spotting and irregular bleeding initially, but such irregularities may reduce over time, and some users may experience a pause in bleeding with these methods.^45–47^ The copper IUD typically results in heavier periods.^48^

Changes to menstrual bleeding patterns represent an important reason for discontinuation and non-use of contraception.^49^ Among those who discontinue contraceptive use in LMICs, more than half do so for method-related reasons, such as side effects and health concerns, and bleeding changes are the major cause of method-related contraceptive discontinuation.^50^ Likewise, the combination of side effects and fear of health concerns is the most commonly reported reason for non-use of contraception among women in LMICs with unmet need for contraception.^51^

On the other hand, some users view the menstrual changes associated with their contraceptive method as having benefits or opportunities. In recent years, the use of hormonal contraception to reduce, control, or pause menstruation, based on personal preference rather than an identified medical need, has gained in popularity, particularly in Europe and North America. The acceptability of contraceptive-induced bleeding changes, however, varies substantially across populations and individuals, and limited evidence exists outside of Europe and North America.^49^ One recent study found that 65% of women in Burkina Faso and 40% of women in Uganda would use a method that pauses bleeding.^52^ Adolescents and young adults may be especially interested in limiting or controlling the timing of menstruation with hormonal contraception.^53,54^

Various factors may contribute to an individual’s preferences for or willingness to tolerate changes to normal bleeding patterns, including age, region, marital or cohabitation status, previous use of hormonal contraception, experience of menstruation, desire to prevent pregnancy and individual and cultural attitudes and beliefs about menstruation.^49^ For instance, some users believe abnormal bleeding patterns lead to infertility and reduced and/or paused bleeding allows “bad” or “dirty” blood to build up in the body.^49,54^

Contraceptive-induced menstrual changes affect people in many of the same ways menstruation does, with both positive and negative impacts on daily life.^49^ Many of these impacts are social constructs and/or personal preferences and thus vary across cultures and among individuals. Menstruation and bleeding can be welcomed if seen as healthy and fertile, a sign of not being pregnant, and a wanted break from chores or sex. On the other hand, bleeding can limit desired engagement in school, work, household, social and religious activities. In many cultures, sexual activity during menstruation is not accepted and contraceptive users may fear prolonged bleeding will cause friction in their relationships and that their partners will seek extramarital relationships.^55,56^ In some cultures, people are expected to socially isolate during menstruation, which further limits their daily activities and may even expose them to harms.^57^ In addition, the management of the bleeding itself can be a challenge, especially when menstrual products are inaccessible, unaffordable, and/or low-quality.^58^ If menstrual or contraceptive-induced bleeding is accompanied by pain, it is even more likely to be seen as undesirable.^49,54^

Improved contraceptive counselling is a recommended strategy for reducing contraceptive discontinuation due to side effects including menstrual changes.^59^ A counselling tool that uses the mnemonic “NORMAL” provides simple messages for providers to build client understanding about contraceptive-induced bleeding changes and reassure them about typical bleeding changes. The tool prompts providers to address myths and misconceptions, and counsel on available treatments for bleeding changes seen as problematic and the benefits of reduced or no bleeding.^60^

### Fertility awareness methods of contraception

Couples have used knowledge of the menstrual cycle and ovulation as a means of preventing pregnancy for nearly a century. Modern fertility awareness methods of contraception, including the Standard Days Method (SDM), the Two Day Method (TDM), the Lactational Amenorrhea Method (LAM), and the “DOT” app, have been shown to have typical use effectiveness rates commensurate with other modern methods of contraception.^61,62^ These methods expand the range of contraceptive options, contribute to improved understanding of biology and fertility, and in the case of SDM and TDM, can also be used in pregnancy planning.^61^

### Perimenopause and menopause

Perimenopause and menopause are unfortunately neglected within both MH and SRHR. With age, hormonal changes cause menstrual cycles to decrease in frequency and eventually cease, corresponding to the decline of fecundity. But even in perimenopause, many individuals are still fecund.^63^ Many pregnancies and births after age 40 are unplanned; data from recent Demographic and Health Surveys indicate the percentage of recent births not wanted among women 45–49 years old was over 40% in Burundi, Malawi and Uganda and above 20% in Cameroon, Indonesia, Senegal and Tanzania.^64^

The hormonal changes of perimenopause can result in several side effects relevant to MH and SRHR including less frequent and less predictable menstrual bleeding and vaginal dryness and/or discomfort during sexual intercourse. Trained health care providers can offer medical and self-care treatments for these and other perimenopausal symptoms.^65^ And while they may no longer be of reproductive age, many post-menopausal individuals continue to have sexual relations and thus continue to be at risk for sexually transmitted infections and gender-based violence.^66^

## Sociocultural linkages between MH and SRHR

Important sociocultural linkages connect the MH and SRHR fields and are reflected in their joint objective to support health, well-being, and equitable access to educational, economic, professional and social opportunities for all people.

### Experience of puberty and menarche

How a child experiences puberty and menarche can affect future reproductive health. Puberty is a time of complex biological, emotional and social changes. The start of menstruation marks a major change. For some children, menarche can be a moment of pride as they enter womanhood but, for others, it can lead to fear, shame and isolation. This is especially true when they experience their first menstruation without any prior knowledge or understanding of what it is or how to manage it, as is the case for many children in LMICs.^67–69^ Studies have shown that children who were better prepared for menstruation had more positive feelings about it.^70,71^

Lack of knowledge and shame about the menstrual cycle can lead to unhygienic and otherwise negative practices.^7,72,73^ This lack of body literacy also contributes to a feeling of lack of bodily control and/or bodily shame, especially in regards to sexuality and reproduction, and may impact a child’s future ability to negotiate safe sex and other reproductive health issues.^10,13,74^ Conversely, providing education about the menstrual cycle may improve reproductive health outcomes. A study in Nepal found that women with stronger understanding of the fertile window within the menstrual cycle were 1.7 times more likely to be using a family planning method.^75^

### Gender norms and equity

Gender norms offer another area where the efforts of the MH and SRHR fields overlap. In many settings, it is during puberty and at menarche that the familial, societal and cultural expectations placed on girls and boys diverge, with girls expected to refrain from leaving the house and to take on more household responsibilities. Boys, meanwhile, are often allowed to continue moving about more freely.^11,76^ Beliefs that menstruation is “dirty” or should be kept hidden contribute to harmful gender norms and specifically, expectations of behaviour restrictions among girls and women, especially during menstruation. Adherence to these restrictive norms may also be self-imposed, as menstruators worry about their ability to contain menstrual leaks and odour, especially if they are unable to access the resources needed to confidently manage their bleeding.^77,78^

These menstruation-related norms are closely linked to puberty and sexual maturation. Behaviour restrictions may also be imposed paternalistically, seen by those implementing them as a means of protecting women and girls, such as by restricting their interactions with men beyond the family and thus protecting them from potential sexual advances and harassment.^79,80^ Restrictive gender norms are associated with SRHR outcomes such as lower likelihood to use contraception^81^ and exposure to intimate partner violence,^82^ among others.^76^

Both MH and SRHR implementers employ strategies for changing social and gender norms to help women and girls engage more fully and on an equal footing with boys and men. Early adolescence – during the pubertal transition and menarche – is recognised as an important time to address harmful gender norms.^11,83^ Interventions to empower women and girls in LMICs have often, though not consistently, been associated with several positive reproductive health-related outcomes,^84^ as have gender-transformative interventions targeting men.^85^

### Education

Evidence from Malawi and other LMICs indicates that girls miss or do not actively participate in school because of challenges related to managing their menstruation.^8,86^ Research on MH interventions, however, shows that they have yielded mixed results in increasing school attendance.^87,88^ One potential reason for this discrepancy is that girls leave school around menarche for various reasons, not all of which can be addressed by improved access to menstrual health resources. Many of these reasons remain linked to puberty and menstruation, such as menstrual pain, perceptions of increased sexual vulnerability,^68^ and parental and cultural influences that discourage girls from attending school especially when menstruating.^89^

Education is a human right and keeping girls and all children in school is a shared objective of the MH and SRHR fields. Evidence demonstrates that the longer a girl stays in school, the more likely she is to use contraception, including condoms; delay marriage and first birth; and use prenatal care and have healthier babies.^90–92^ Education empowers people to engage in decision-making and wage-earning and can influence gender norms.^83^

### Gender-based violence and transactional sex

MH issues also potentially affect SRHR outcomes related to gender-based violence and transactional sex. Where WASH facilities are at a distance from residences and taboos around menstruation are strong, women and girls may prefer to go to the toilet at night, when the darkness provides some level of privacy. Unfortunately, evidence suggests that this places them at risk of harassment and sexual assault.^80,93^

In addition, a few studies, all in Kenya, have found that some adolescents engaged in transactional sex in order to obtain sanitary pads or the funds to purchase them.^68,78,94^ Transactional sex can expose girls and women to HIV infection and violence.^95^

## Recommendations for MH and SRHR integration

Given the connections between MH and SRHR, increased integration of the fields has the potential to help achieve their common goals. By ignoring MH, the SRHR field risks missing opportunities to improve health and well-being and to increase gender equality. Integrating MH and SHRH more intentionally could improve efforts to reduce STI and HIV rates, prevent unintended pregnancies, keep girls in school, reduce gender-based violence, and support women’s participation in the workforce. In addition, supporting MH interventions among children and very young adolescents could help address the root causes of and potential contributing factors to negative SRHR outcomes, including the potential to address gender norms while they are more fluid.^11^

We have provided several recommendations for integrating MH and SRHR programming in the sections above. Additional cross-cutting steps which may be relevant for MH and SRHR practitioners, as well as for policy makers, researchers and donors, to better connect these fields and serve their communities include:

- *Explicitly incorporate MH into SRHR*, including in descriptions and definitions of SRHR, as well as in SRHR policies, guidance, and programmes. In addition*,* government agencies, implementing organisations, funding groups and others working on SRHR should build their capacity in MH.
- *Collect more MH data and evaluate integrated MH-SRHR programmes*. While several large-scale demographic surveys and longitudinal studies of adolescents have recently started collecting data on menstrual health,^12,96,97^ further work is needed to refine and incorporate menstrual health indicators for broader use.^98^ Also, efforts are needed to design, pilot, evaluate, and implement programmes and policies that better integrate MH and SRHR.
- *Strengthen implementation of comprehensive sexuality education (CSE)* that reaches a wide age range of children and adolescents and covers a broad spectrum of age-appropriate topics. Well-developed and well-implemented CSE programmes not only allow children to experience puberty and menstruation with a clear understanding of what is happening to their bodies, but can also reduce taboos and stigma. Engagement of parents, caregivers and community stakeholders is also critical and can enhance the impact of CSE programmes.^99^
- *Support health care providers, including community health workers, to discuss menstruation, menstrual disorders*, *contraception-induced menstrual changes and management options during provider-client counselling and health education sessions*. Providers should have the knowledge and resources to diagnose and treat menstrual disorders and the skills to advise clients about contraceptive-induced menstrual changes, including providing support for self-care.^100^ Family planning providers should be trained to deliver messages like those in the NORMAL job aid,^60^ including the benefits of reduced or no bleeding, as well as to distribute menstrual products and discuss menstruation and fertility awareness.

## Conclusion

Menstrual health is part of the continuum of sexual and reproductive health across the life course. The fields of MH and SHRH have shared values and goals and offer numerous opportunities for linkages that could improve outcomes for both. Supporting individuals to manage their menstruation and MH provides them with dignity and is a human right. As a community committed to health, well-being, and gender equality, SRHR practitioners must embrace MH as an integral part of advocacy, policy and programming.

## Author contributions

LCW, KHR, JR, SF, GN, and RLC conceptualised the article, provided critical review and revisions, and approved the final version. LCW drafted the article. ACLM provided critical review and revisions leading to substantial changes in content and approved the final version.
